# Alteration in the Expression of Cytochrome P450s (CYP1A1, CYP2E1, and CYP3A11) in the Liver of Mouse Induced by Microcystin-LR

**DOI:** 10.3390/toxins7041102

**Published:** 2015-03-30

**Authors:** Bangjun Zhang, Yang Liu, Xiaoyu Li

**Affiliations:** College of Life Science, Henan Normal University, Xinxiang 453007, Henan, China; E-Mails: 041129@htu.cn (B.Z.); liuyang6368@126.com (Y.L.)

**Keywords:** MCLR, mouse, cytochrome P450, mRNA level, protein content, enzyme activity

## Abstract

Microcystins (MCs) are cyclic heptapeptide toxins and can accumulate in the liver. Cytochrome P450s (CYPs) play an important role in the biotransformation of endogenous substances and xenobiotics in animals. It is unclear if the CYPs are affected by MCs exposure. The objective of this study was to evaluate the effects of microcystin-LR (MCLR) on cytochrome P450 isozymes (CYP1A1, CYP2E1, and CYP3A11) at mRNA level, protein content, and enzyme activity in the liver of mice the received daily, intraperitoneally, 2, 4, and 8 µg/kg body weight of MCLR for seven days. The result showed that MCLR significantly decreased ethoxyresorufin-*O*-deethylase (EROD) (CYP1A1) and erythromycin *N*-demthylase (ERND) (CYP3A11) activities and increased aniline hydroxylase (ANH) activity (CYP2E1) in the liver of mice during the period of exposure. Our findings suggest that MCLR exposure may disrupt the function of CYPs in liver, which may be partly attributed to the toxicity of MCLR in mice.

## 1. Introduction

Microcystins (MCs) are the most commonly detected cyanobacterial toxins produced mainly by the cyanobacterium *Microcystis* in eutrophic lakes, ponds, and rivers throughout the world [1]. Currently, MCs have over 80 known structural variants, of which microcystin-LR (MCLR) is the most toxic variant found in the aquatic environment [2]. These toxins have been shown to cause severe harmful effects in aquatic organisms and humans. The toxicity mechanism of MCs is attributed to their activity as potent inhibitors of serine/threonine protein phosphatases 1 (PP1) and PP2A in liver [3]. This inhibition leads to the hyperphosphorylation of cytoskeleton protein and destroys its components, which induces hepatocyte deformation, dissociation, and necrosis, and ultimately causes intrahepatic hemorrhage and death [4,5]. Additionally, accumulating evidence indicates that oxidative stress also plays an important role in the toxicity of MCs [6,7,8]. The induction of oxidative stress by microcystins was found in mouse [9], rat [10,11], fish [12], frog [13], and cell lines [14,15]. Weng *et al.* [16] reported that reactive oxygen species (ROS) played a critical role in MCLR-induced hepatocyte apoptosis and liver injury in mice. Žegura *et al.* [17] found that the genotoxicity of MCLR was mediated by ROS in human hepatoma HepG2 cells. Moreover, MCLR-induced ROS also caused cytoskeletal disruption in the hepatocytes of common carp (*Cyprinus carpio* L.) [18].

Cytochromes P450s (CYPs) belong to a superfamily of heme monooxygenases that catalyze oxidation of endobiotic lipids and steroidal hormones, as well as numerous xenobiotic chemicals, including drugs, carcinogens, and environmental contaminants [19,20]. Among the various CYP isoenzymes, CYP1, CYP2, and CYP3 are three major families of the CYP superfamily that are involved in metabolism and biotransformation a wide variety of xenobiotic chemicals [19]. CYP1A1 is responsible for the metabolism or activation of many kinds of procarcinogens, such as polycyclic aromatic hydrocarbons (PAH) [21]. CYP2E1 has been shown to be involved in the metabolism of many low-molecular toxic hydrophobic chemicals, such as benzene, CCl_4_, nitrosamines, thioacetamide, and pyridine [22,23]. Particularly, CYP3A accounts for 25%–35% of the total cytochrome P450 present in adult human liver or rat liver, which is involved in oxidative metabolism of a variety of clinically used drugs [24]. It has been reported that MC exposure significant induces CYP1A2 expression in mouse by using the DNA microarray, suggesting that CYP1A2 might be activated by MC [25]. Guo *et al.* [26] found that okadaic acid (analog of MC) was a substrate for human cytochromes CYP3A4 and CYP3A5 *in vitro*. Moreover, transcription of CYP3A65 was found to be significantly altered by MC-exposure of zebrafish liver [27]. However, to the best of our knowledge, there is no report about the effects of MCs on the three main CYP450 subfamilies, CYP1A, CYP2E, and CYP3A, simultaneously in mouse liver at levels of transcription, protein content, and enzyme activity.

The aim of our study was to investigate the response of phase I metabolism enzymes, such as CYP1A1, CYP2E1, and CYP3A11, in mouse liver following 7 d of MCLR-exposure. The enzyme activity of CYP1A1 was represented by ethoxyresorufin-*O*-deethylase (EROD), CYP2E1 by aniline hydroxylase (ANH), and CYP3A11 by erythromycin *N*-demthylase (ERND). The study may provide insight into the biochemical mechanism related to the toxicity of MCLR in mice.

## 2. Result

### 2.1. Effects of MCLR on mRNA Level, Protein Content, and Enzymic Activity of CYP1A1 in Mouse Liver 

**Figure 1:**
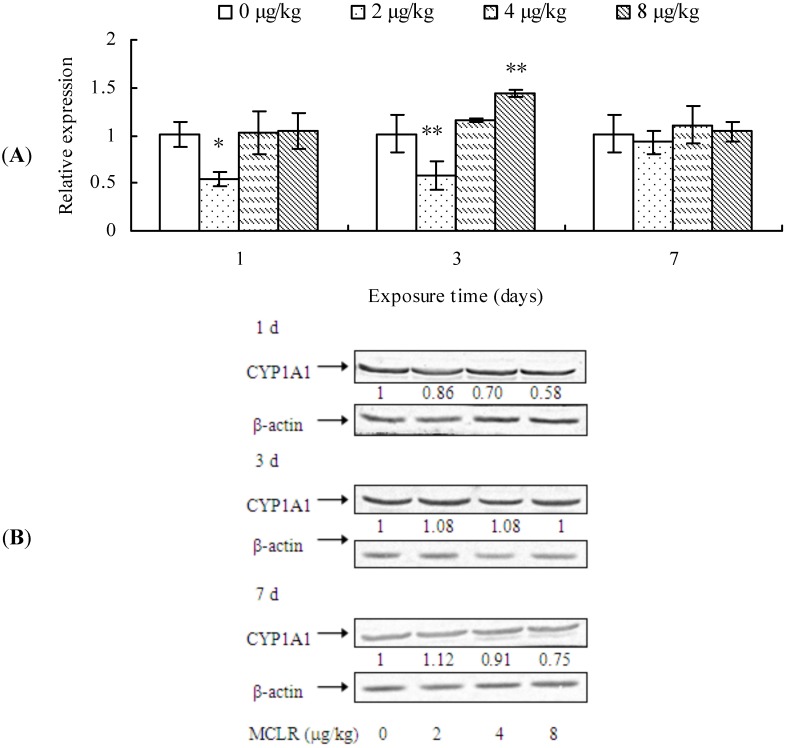

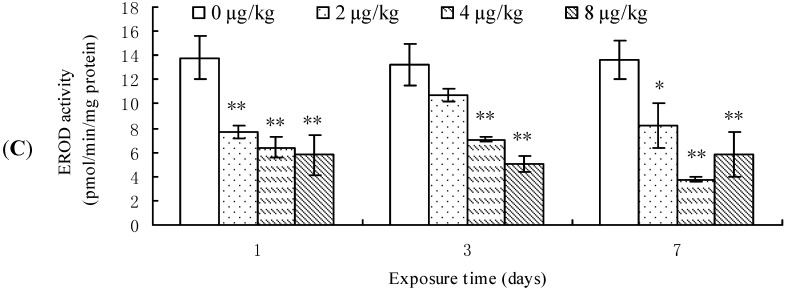
Cytochrome P450 1A1 (CYP1A1) mRNA level (**A**); protein content (**B**); and ethoxyresorufin-*O*-deethylase (EROD) activities (**C**) in mouse liver. Asterisks denote a response that is significantly different from the control (* *p <* 0.05, ** *p <* 0.01). CYP1A1 protein contents were normalized to β-actin followed by analysis of the relative intensity. The values indicated the fold change compared to the control (0 μg/kg).

CYP1A1 mRNA level, protein content, and EROD activity in mouse liver are demonstrated in Figure 1. CYP1A1 transcription was significantly down-regulated in the mouse liver from 2 μg/kg treatment group after 1 day of exposure when compared to the control group. However, after 3 days of treatment, it was up-regulated in 8 μg/kg group, although it was still down-regulated in 2 μg/kg group. Nevertheless, at the end of MCLR-exposure, CYP1A1 transcription remained unchanged in all treatment groups (Figure 1A). A dose-dependent decrease of CYP1A1 protein content was observed after 1 day of exposure, while it remained unchanged at 3 days. However, after 7 days of exposure, it exhibited a slight increase in the 2 μg/kg group, but a decrease in the 4 and 8 μg/kg groups (Figure 1B). EROD activities in all treatment groups decreased significantly throughout the experimental period in comparison to that of control groups (Figure 1C).

Our results showed that MCLR exposure significantly decreased CYP1A1 transcription level at 2 μg/kg group. However, CYP1A1 protein content showed a dose-dependent decrease after 1 or 7 days of MCLR exposure. Moreover, MCLR also inhibited the EROD activities of mouse liver.

### 2.2. CYP2E1 Transcription, Protein Content, and ANH Activity

**Figure 2:**
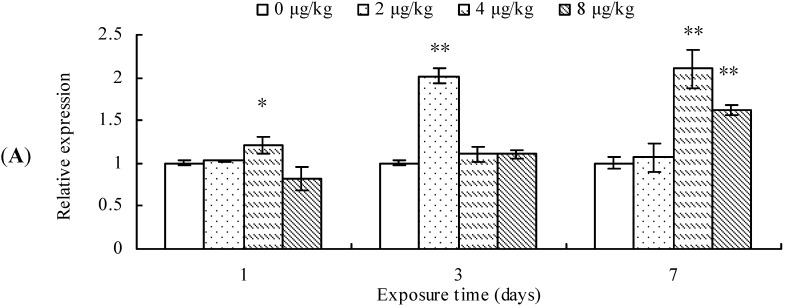

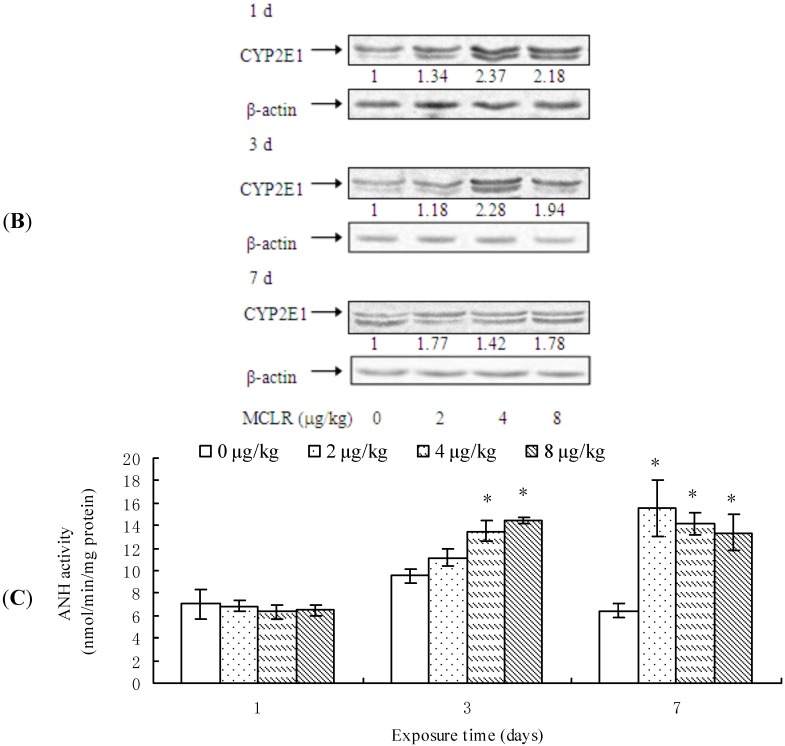
Cytochrome P450 2E1 (CYP2E1) mRNA level (**A**); protein content (**B**); and aniline hydroxylase (ANH) activities (**C**) in mouse liver. Asterisks denote a response that is significantly different from the control (* *p <* 0.05, ** *p <* 0.01). CYP2E1 protein contents were normalized to β-actin followed by analysis of the relative intensity. The values indicated the fold change compared to the control (0 μg/kg).

CYP2E1 transcription was significantly up-regulated in the mice exposed to 4 μg/kg of MCLR for 1 day, 2 μg/kg MCLR for 7 days, or 4 and 8 µk/kg MCLR for 7 days (Figure 2A). Furthermore, CYP2E1 protein content was significantly increased in all MCLR-treated groups at 1, 3, and 7 day (Figure 2B). ANH activities increased in 4 and 8 μg/kg of MCLR groups after 3 days of exposure. After 7 days of exposure, ANH activities were significantly increased in all treatment groups (Figure 2C).

In short, MCLR promoted the expression of CYP2E1.

### 2.3. CYP3A11 mRNA Level, Protein Content, and ERND Activity

**Figure 3:**
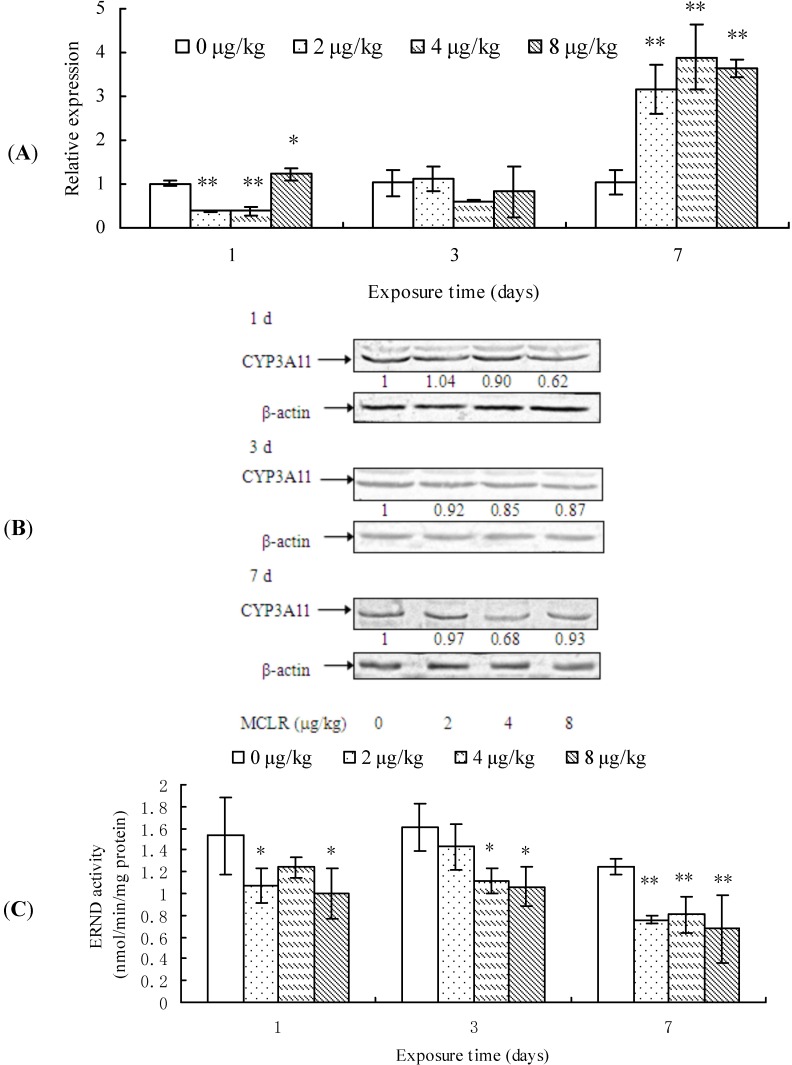
Cytochrome P450 3A11 (CYP3A11) mRNA level (**A**); protein content (**B**); and erythromycin *N*-demthylase (ERND) activities (**C**) in mouse liver. Asterisks denote a response that is significantly different from the control (* *p <* 0.05, ** *p <* 0.01). CYP3A11 protein contents were normalized to β-actin followed by analysis of the relative intensity. The values indicated the fold change compared to the control (0 μg/kg).

After 1 day of MCLR exposure, CYP3A11 transcription was remarkably suppressed in 2 and 4 µg/kg groups, while promoted in 8 µg/kg group, but it remained unchanged after 3 days. However, at the end of exposure, it was significantly up-regulated again in all treatment groups, when compared to the control group (Figure 3A). The protein content of CYP3A11 decreased in a dose-dependent manner in almost all treatment groups from 1 to 7 days of exposure in comparison with control groups (Figure 3B). In a similar way, ERND activity decreased in almost all treatment groups throughout the period of test (Figure 3C).

Our results reveal that MCLR exposure basically suppresses CYP3A11 expression, except for the transcriptional promotion at the end of exposure.

## 3. Discussion

CYP enzymes play a key role not only in metabolizing endogenous substances, but also in biotransformation of xenobiotics or environmental pollutants [28]. Many studies showed that CYP activity can reflect the presence of environmental pollutants [29]. Alterations in CYP expression or enzymic activity in various organisms by many environmental contaminations have been reported; for example, heavy metals [30,31], 2,3,7,8-tetrachlorodibenzo-p-dioxin (TCDD) [32,33], and dexamethasone [34]. It was reported that *Microcystis aeruginosa* 7820 decreased the levels of cytochrome b5 and cytochrome P450 in mouse [35]. Recent studies also showed that MCs induced the change of CYP3A65 transcription in zebrafish [27] and CYP1A2 in mice [25]. The present study aimed to evaluate an integral situation of three major CYP subfamilies, CYP1A1, CYP2E1, and CYP3A11, simultaneously in mouse liver following intraperitoneal (i.p.) exposure of MCLR for seven days.

It is well known that CYP1A1-dependent EROD activity in organisms has been used extensively as a biomarker of environmental toxicants, such as polycyclic aromatic hydrocarbons (PAH), polychlorated biphenyl (PCB) or polychlorodibenzo-*p*-dioxine (PCDD), due to the fact that EROD activity in animals can be significantly induced by these toxicants [36,37,38,39,40]. However, it was reported that other contaminations, such as heavy metals, could also inhibit EROD activity in HepG2 cells [41], fish hepatoma cells PLHC-1 [42], or Hepa1c1c7 cells [31]. In the present study, MCLR significantly inhibited the EROD activities in mouse liver. However, we also found that MCLR suppressed CYP1A1 transcription only in 2 µg/kg group at 1 and 3 days of exposure. Moreover, the change tendency of CYP1A1 protein contents was not consistent with that of EROD activity. These results suggest that the inhibition of EROD activity may be related with MC toxicity at post-transcription or post-translational level.

CYP2E1 is a key member of CYP superfamily and plays an important role in metabolizing low-molecular hydrophobic chemicals [43]. Moreover, it can convert molecular oxygen to highly reactive compounds, including superoxide anion radical, singlet oxygen, hydrogen peroxide and hydroxyl radical, which may lead to DNA damage and carcinogenesis [44,45]. It was reported that many CYP2E1 substrates, such as acetone, alcohol, benzene, and pyridine, could induce the CYP2E1-associated enzyme activities, such as *N*-nitrosodimethylamine *N*-demethylase, *p*-nitrophenol hydroxylase, and aniline 4-hydroxylase in rat and rabbit liver [22,43,46]. Moreover, CYP2E1 inducers can increase the toxicity of toxic and/or carcinogenic chemicals when they are added prior to the exposure of chemicals [22]. For example, acetone promoted the hepatotoxicity of cisplatin in mice [47]. Our results showed that MCLR significantly increased CYP2E1 mRNA level, protein content, and ANH activity in mouse liver. This was in accordance with the report by Nong *et al.* [7], in which they found that HepG2 cells treated with MCLR showed a significant increase in CYP2E1 mRNA level. They also found that CYP2E1 inhibitors chlormethiazole and diallyl sulphide decreased both ROS generation and cytotoxicity of MCLR in HepG2 cells. Therefore, we suppose that the induction of CYP2E1 activity may increase the generation of ROS in mouse liver.

CYP3A subfamily is the most abundant and important group of CYP enzymes, which participates in metabolizing not only numerous xenobiotics but also endogenous compounds, such as steroid hormones and bile acids [48,49]. In this study, we investigated the effects of MCLR exposure on CYP3A11 expression in mouse liver. Our results indicated that MCLR increased CYP3A11 mRNA level in mouse liver after seven days of MCLR exposure. However, CYP3A11 protein contents in mouse liver decrease after MCLR exposure. It has been reported that CYP3A is the major enzyme catalyzing erythromycin *N*-demethylation in mouse liver [50] and they suggest that ERND activity might be used as a biomarker of CYP3A expression. The present results indicate that MCLR exposure inhibits ERND activity in all treatment groups. The decreased ERND activity in mice may alter the metabolism activities of CYP3A to endogenous substances, such as testosterone, which may disturb the normal biological function of mouse.

The inhibition of CYP1A1 and CYP3A11 activities by MCLR exposure suggest that these enzymes may not be involved in the metabolism of MCLR in mouse liver. The increase in CYP2E1 activity indicates that CYP2E1 is possibly involved in MCLR metabolism. It has been reported that ROS play a role in the decrease of CYP450 activity *in vivo* and *in vitro* [51]. Elbekai *et al.* [51] found that As^3+^, Cd^2+^, and Cr^6+^ could inhibit CYP1A1 activity in Hepa 1c1c7 cells and pretreatment with the antioxidant *N*-acetylcysteine (NAC) completely abrogated the inhibition. Galal and Souich [52] reported that hypoxia-induced ROS decreased the total hepatic P450. In the present study, the induction of CYP2E1 may increase the ROS generation, which may be attributed to the inhibition of CYP1A1 and CYP3A11 activities. More, the induction of CYP2E1 may be partly responsible for the toxicity of MCLR.

## 4. Materials and Methods

### 4.1. Reagents

MC-LR with a purity of more than 95% was purchased from Taiwan Algal Science Inc., Taoyuan, Taiwan. Total RNA Isolation Kit was purchased from Beijing TransGen Biotech Co., Ltd., Beijing, China. First-Strand cDNA Synthesis Kit was purchased from Beijing Cowin Biotech Co., Ltd., Beijing, China. Antibodies against CYP1A1, CYP2E1, CYP3A11, and β-actin were obtained from Proteintech Group, Wuhan, China. Other chemicals were obtained from commercial source and were of analytical grade.

### 4.2. Animals and MCLR-Exposure

Male Kunming (KM) mice (20–22 g) were obtained from Experimental Animal Centre of Henan Province, China. They were temporarily reared in the animal house with wood shavings as bedding under controlled conditions, with a 12-h light/dark cycle and temperature (21–22 °C) for at least one week before the experiment. Mice were allowed access to a standard rodent pellet diet and water *ad libitum*. All experimental procedures were performed according to the guidelines in the China Law for Animal Health Protection and Instructions for Granting Permit for Animal Experimentation for Scientific Purposes (Ethics approval No. SCXK (YU) 2005-0001).

After acclimatization, the mice were randomly divided into 4 groups (15 mice in each group), in which 3 groups were taken as the MCLR-treatment groups and one as a control group. Mother solution of MCLR (100 μg/mL) was obtained by dissolving the MCLR into sterilized saline solution. The mice received an intraperitoneal (i.p.) injection of MCLR daily for 7 days at a dose of 2, 4, or 8 µg/kg body weight, respectively. The doses based on our preliminary research in which the LD_50_ of MCLR to mice was 70 µg/kg body weight. The control group was injected with the same volume of 0.9% saline solution. After being anesthetized, 5 mice in each group were sacrificed at 1, 3, and 7 days of exposure, respectively, and the liver from control and exposed animals were quickly removed and stored at −80 °C for the following biochemical assays.

### 4.3. RNA Extraction and Real Time PCR

Total RNA in mouse liver from each group was extracted using *TransZol* kit (Beijing TransGen Biotech Co., Ltd., Beijing, China), according to the manufacturer’s protocol and RNA concentration was quantified with a Nanodrop spectrophotometer. The first strand cDNA was synthesized from 1 µg of RNA by using First-Strand cDNA Synthesis Kit (Beijing Cowin Biotech Co., Ltd., Beijing, China). Quantitative real-time polymerase chain reaction (q-PCR) was used to determine the expressions of genes, such as CYP1A1, CYP2E1, CYP3A11, and β-actin by using UltraSYBR Mixture (Beijing Cowin Biotech Co., Ltd., Beijing, China). The primers of q-PCR are described in Table 1. The expression of individual genes was performed using the ABI 7500 Real-time PCR system. The amplifications for all reactions were performed as following: 10 min 95 °C, followed by 40 cycles of 30 s at 95 °C and 40 s at 60 °C. Results were calculated using the relative 2^−ΔΔCt^ method [53].

**Table 1:** Primers for amplification of the listed genes by real-time PCR.

Gene	Forward (5' to 3')	Reverse (5' to 3')
CYP1A1	GGTTAACCATGACCGGGAACT	TGCCCAAACCAAAGAGAGTGA
CYP2E1	AAGCGCTTCGGGCCAG	TAGCCATGCAGGACCACGA
CYP3A11	ATTCCTGGGCCCAAACCTCTGCCA	TGGGTCTGTGACAGCAAGGAGAGGC
β-actin	CACCCGCGAGCACAGCTTCTTT	TTGTCGACGACCAGCGCAGCGATA

### 4.4. Western Blot Analysis

The liver was homogenized in 1:10 (*w*/*v*) RIPA buffer containing protease inhibitor cocktail (Beijing Solarbio Science & Technology Co., Ltd., Beijing, China). Samples were then centrifuged at 10,000× *g* for 10 min at 4 °C and supernatants were collected. The concentration of protein was estimated by using BCA protein assay kit (Beyotime Institute of Biotechnology, Haimen, China). Protein (30 µg) was mixed with 5× loading buffer (Beyotime Institute of Biotechnology, Haimen, China), boiled for 5 min, then separated by 10% SDS-PAGE, and finally transferred to a nitrocellulose membrane. The membrane was blocked for 1 h at room temperature in a blocking buffer containing 5% nonfat dry milk, 0.05% (*v*/*v*) Tween 20 in a Tris-buffered saline (25 mM Tris-HCl buffer, pH 7.5 and 0.15 M sodium chloride). After blocking, the membrane was incubated with rabbit polyclonal antibodies of CYP1A1, CYP2E1, CYP3A11, and β-actin overnight at 4 °C and secondary antibody (goat anti-rabbit IgG conjugated to alkaline phosphatase, 1:1000, Beyotime Institute of Biotechnology, Haimen, China) at room temperature for 2 h. The membrane was then washed with TBS 3 times for 10 min. The protein signal of immunoblot analysis was visualized by using NBT/BCIP system. The figures presented were representatives of at least three independent assays. The specific bands were analyzed by the ImageJ Image Processing Program (National Institutes of Health, Bethesda, MD, USA, http://rsb.info.nih.gov/ij).

### 4.5. Preparation of Microsomes and Enzyme Activity Assay

Microsomes of mouse liver were prepared according to the method as previously described [54]. CYP1A1 activity in the microsome was determined by ethoxyresorufin-*O*-deethylase (EROD) activity according to Sinal *et al.* [55] and CYP2E1 activity was measured by aniline hydroxylase (ANH) activity based on the method of Imai *et al.* [56]. CYP3A11 activity represented by erythromycin *N*-demthylase (ERND) activity was assessed by quantitation of formaldehyde release using the Nash reagent [57]. The content of total protein was determined by the method of Bradford [58] by using albumin as a standard.

### 4.6. Statistical Analysis

All results were analyzed by one-way analysis of variance (ANOVA) and Dunnett’s multiple comparison tests with SPSS 11.5 software (SPSS Inc., Chicago, IL, USA, 2003). Differences between means were determined at the *p* < 0.05 or *p* < 0.01 levels for all analyses and indicated with * and **, respectively.

## 5. Conclusions

In conclusion, the results of this study indicated that MCLR inhibited the activities of CYP1A1 and CYP3A11 in mouse liver. The results also showed that MCLR increased the CYP2E1 activity, which may increase the ROS generation in mouse liver. This result may provide new insight in the mechanism of ROS generation in mice after MCLR exposure. However, further studies are needed to fully understand the relationship between CYP2E1 enzyme and MCLR toxicity in mouse.
